# Survival analysis and functional annotation of long non‐coding RNAs in lung adenocarcinoma

**DOI:** 10.1111/jcmm.14458

**Published:** 2019-06-18

**Authors:** Abbas Salavaty, Zahra Rezvani, Ali Najafi

**Affiliations:** ^1^ Division of Biotechnology, Faculty of Chemistry, Department of Cell and Molecular Biology University of Kashan Kashan Iran; ^2^ Molecular Biology Research Center, Systems Biology and Poisonings Institute Baqiyatallah University of Medical Sciences Tehran Iran

**Keywords:** bioinformatics, functional annotation, lung adenocarcinoma, prognostic lncRNAs, systems biology

## Abstract

Long non‐coding RNAs (lncRNAs) are a subclass of non‐protein coding transcripts that are involved in several regulatory processes and are considered as potential biomarkers for almost all cancer types. This study aims to investigate the prognostic value of lncRNAs for lung adenocarcinoma (LUAD), the most prevalent subtype of lung cancer. To this end, the processed data of The Cancer Genome Atlas LUAD were retrieved from GEPIA and circlncRNAnet databases, matched with each other and integrated with the analysis results of a non‐small cell lung cancer plasma RNA‐Seq study. Then, the data were filtered in order to separate the differentially expressed lncRNAs that have a prognostic value for LUAD. Finally, the selected lncRNAs were functionally annotated using a bioinformatic and systems biology approach. Accordingly, we identified 19 lncRNAs as the novel LUAD prognostic lncRNAs. Also, based on our results, all 19 lncRNAs might be involved in lung cancer‐related biological processes. Overall, we suggested several novel biomarkers and drug targets which could help early diagnosis, prognosis and treatment of LUAD patients.

**Highlights**

- Nineteen lncRNAs are presented as novel prognostic biomarkers for LUAD.
- The plasma abundance of SNHG6 could be used as a diagnostic and/or prognostic biomarker in LUAD.
- LncRNAs could involve in LUAD development through influencing the hsa04080 KEGG pathway.

## 1 INTRODUCTION

Lung cancer is the number one cause of cancer‐related death among both men and women worldwide.^1^ Non‐small cell lung cancer (NSCLC) accounts for approximately 85% of lung cancer cases.^2^ NSCLC is histologically divided into three subtypes of which lung adenocarcinoma (LUAD) and lung squamous cell carcinoma (LUSC) account for ~50% and ~40% of the cases respectively.^3^ Unfortunately, most of the NSCLC patients are diagnosed at advanced stages and have a very poor prognosis, which consequently results in a low overall survival (OS) rate (15%).^4^, ^5^ In this regard, LUAD is one of the most aggressive and deadliest types of cancer with less than 5 years of OS.^6^ A variety of factors such as cigarette smoking, exposure to second‐hand smoke, air pollution, cooking fumes, asbestos and radon put individuals at the risk of LUAD. Besides, immunologic dysfunction, genetic susceptibility as well as some diseases including asthma and tuberculosis infections would enhance the risk of LUAD.^7^ Additionally, it is reported that the carcinogenesis of LUAD varies between men and women as well as between smokers and never smokers.^8^

Long non‐coding RNAs (lncRNAs) refer to a class of non‐protein coding RNAs that are more than 200 nucleotides and are differentially accumulated in the nucleus and cytoplasm.^9^ LncRNAs play various regulatory roles in the cell including regulation of development, stem cell pluripotency, cell growth and apoptosis and are frequently dysregulated in different cancers.^9^, ^10^ HOTAIR, as an example, is a well‐known oncogenic lncRNA that is up‐regulated in several cancers.^11^ The lncRNA linc00665 has recently been represented as an oncogenic factor in LUAD.^12^ However, there are hundreds of lncRNAs that their exact roles in different cancers are yet to be discovered and/or experimentally validated. RAB6C‐AS1, for instance, is a poorly known lncRNA that is presented as a potential candidate biomarker for prostate and brain cancers but its implications in the carcinogenesis of these cancers are not still neither computationally nor experimentally examined and validated.^13^ LncRNAs are also involved in the tumourigenesis and progression of lung cancer through aberrant regulation of gene expression at the transcriptomic, epigenomic and genomic levels.^14^ Additionally, epigenetic and RNA deregulations are considered as a potential hallmark of LUAD.^15^ Altogether, discovering the functional roles of lncRNAs in LUAD would greatly enhance our knowledge of the aetiology of LUAD and lead to the advent of novel promising biomarkers and drug targets for this deadly disease.

As lncRNAs play essential roles in the progression of different cancers, they have the potential to be used as diagnostic and prognostic biomarkers.^16^ Also, the presence of several circulating transcripts has been reported in the plasma and serum of cancer patients which could be used for diagnostic purposes.^17^ Moreover, different circulatory non‐coding RNAs (ncRNAs) including lncRNAs are being constantly represented as biomarkers for cancer diagnosis, prognosis and monitoring of treatment response.^5^, ^18^ Thus, lncRNAs are potential factors for the prediction of OS and disease‐free survival (DFS) periods of cancer patients. Today, lncRNAs are being regarded as potential diagnostic factors and therapeutic targets for NSCLC.^19^ The lncRNA LINC00578, as an example, is represented as a promising biomarker and therapeutic target for LUAD.^20^ In another study, TG et al introduced three lncRNAs including HCP5, SNHG12 and LINC00472 as potential biomarkers for LUAD management.^21^ Also, several lncRNAs such as LHFPL3‐AS2, LINC01105, LINC00092, LINC00908 and FAM83A‐AS1 have been reported as prognostic factors for LUAD.^22^ Furthermore, the diagnostic value of circulating lncRNAs as plasma signatures for the early detection of lung cancer has been confirmed.^23^

A growing number of computational models are being constantly developed for the identification of lncRNA‐disease associations and characterization of functional roles of lncRNAs in diseases including lung cancer. KATZLDA, as an example, is a robust computational model for the prediction of lncRNA‐disease associations.^24^ In another study, Chen et al proposed a kind of top‐down model. They assumed that similar diseases tend to be associated with functionally similar lncRNAs and accordingly, developed a computational model named LRLSLDA.^25^ Generally speaking, identification of lncRNA‐disease associations is achieved based on two different approaches; using known lncRNA‐disease associations, as in machine learning‐based and network‐based models, or using models based on the known disease‐related genes/miRNAs. Functional similarity calculation method, which is based on the assumption that functionally similar lncRNAs are associated with similar diseases, is commonly applied in both of the aforementioned approaches but usually in combination with other methods.^26^ Various information resources are used for the calculation of lncRNA functional similarity which could be summarized into four categories: lncRNA expression similarity, GO term‐based lncRNA functional similarity, miRNA/mRNA‐lncRNA interaction‐based functional similarity and lncRNA‐disease association‐based functional similarity.^27^ In the current study, a GO term‐based lncRNA functional similarity method was used to functionally interrogate the lncRNA‐LUAD associations. In the context of the prediction of functional roles of lncRNAs in diseases, several computational models have thus far been proposed that could be classified into four major categories, including gene coexpression‐based models, lncRNA‐miRNA/mRNA/protein interaction‐based models, sequence alignment‐based models and integrative features‐based models which incorporates sequence‐derived and experimental features of lncRNAs.^27^

In this study, we applied a coexpression‐based model for the prediction of functional roles of lncRNAs in LUAD. It is frequently reported that lncRNAs are differentially expressed (DE) in cancer tissues.^28^ Also, according to the guilt by association principle, if a gene shows an expression correlation with the expression profiles of a set of genes involved in a specific function, that gene is possibly involved in the same function.^29^ Therefore, identification of the coexpressed genes (CEGs) of DE‐lncRNAs can help functional annotation of lncRNAs in cancer. Moreover, CEGs can have common regulatory sequences and might be interacting partners of the same complex and/or involve in the same pathway.^30^ Actually, dysregulated lncRNAs interact with other macromolecules and consequently drive various cancer manifestations.^31^ Hence, identification of the CEGs of DE‐lncRNAs in cancer assists in the characterization of oncogenic or tumour suppressive functions of lncRNAs and recognition of pathways they are involved in. This is a common methodology that can be used for the functional annotation of poorly known genes in the context of different diseases including cancer.^32^

**Figure 1:**
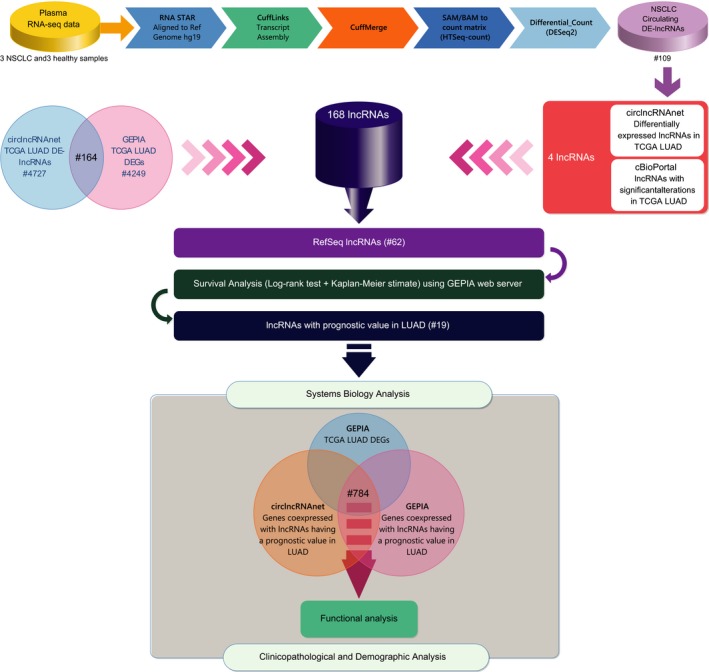
Schematic outline of the research protocol

There are several notable variances in the gene expression profile and molecular features between LUSC and LUAD and consequently different therapeutic strategies and regiments are administrated to these two major subtypes of NSCLC.^33^ Thus, NSCLC studies should precisely target either LUAD or LUSC so as to get more specific results. In this study, we systematically analysed the prognostic value of lncRNAs for LUAD and annotated their functional roles using a bioinformatic and systems biology approach. A schematic outline of the implemented methodology is given in Figure 1.

## 2 METHODS

### 2.1 Data preparation

All of The Cancer Genome Atlas (TCGA) LUAD DE‐lncRNAs (1 < |Log2FC|; adjusted *P*‐value (adjp) < 0.05) were retrieved from the circlncRNAnet (Table S1).^34^ The circlncRNAnet is an integrated web‐based resource for mapping functional networks of long or circular forms of ncRNAs. TCGA LUAD is one of the projects conducted by TCGA Research Network and comprises 483 LUAD tumour samples and 59 normal lung samples. Also, all TCGA LUAD DEGs were obtained from GEPIA (Table S2).^35^ GEPIA is a web server specialized for analysing the RNA‐seq data of 9736 tumours and 8587 normal samples from the TCGA and the GTEx projects. In the context of the differential expression analysis of genes in LUAD, GEPIA has added 288 normal lung samples from GTEx projects to the normal samples of TCGA LUAD so as to make a higher balance between the number of normal and cancer samples. Then, common DE‐lncRNAs between circlncRNAnet and GEPIA with the same expression dysregulation (either over‐ or underexpression) in both databases were selected. Moreover, a total of six plasma RNA‐seq data samples, including three normal and three NSCLC plasma samples, were retrieved from the PRJNA286036 study at the European Nucleotide Archive and analysed using an Australian Galaxy server (GVL QLD, GVL 4.0.1; https://galaxy-qld.genome.edu.au/galaxy).

### 2.2 RNA‐seq data analysis

We applied the following pipeline with this exact sequence of steps for analysing the plasma RNA‐seq data obtained from the European Nucleotide Archive; reads were mapped to the hg19 reference genome using STAR^36^; lncRNA transcripts were assembled using Cufflinks^37^ according to the GTF (UCSC compatible) GRCh37/hg19 Version 5.0 full database annotation file downloaded from the LNCipedia^38^, ^39^; all Cufflinks' GTF output files and the LNCipedia GTF file were merged using Cuffmerge^37^; read counts were calculated using the SAM/BAM to count matrix tool based on the HTSeq code^40^; Differential_Count tool was used to analyse the matrix of the read counts for differentially expressed genes according to the DESeq2 method^41^; the Benjamini‐Hochberg method was used for multiple hypothesis correction; finally, DE‐lncRNAs with adjp under 0.05 were extracted. As the documents of PRJNA286036 study have not mentioned the exact NSCLC subtype of cancer samples, the extracted lncRNAs were queried across circlncRNAnet TCGA LUAD and cBioPortal^42^, ^43^ TCGA LUAD, Provisional, datasets to find and select the ones with significant alterations in LUAD.

### 2.3 Data filtration

All of the prepared data were filtered so as to make our downstream analyses more specific. First, the list of lncRNAs selected from the intersection of data retrieved from circlncRNAnet and GEPIA databases was combined with the list of lncRNAs outputted from the RNA‐seq data analysis. This combined list was named as the gene library (gene library = (circlncRNAnet DE‐lncRNAs ⋂ GEPIA DEGs) ⋃ (NSCLC plasma DE‐lncRNAs ⋂ LUAD altered/DE‐lncRNAs)). Then, the lncRNAs without RefSeq sequences in the gene library were filtered out in order to lay a firm foundation for our downstream analyses. To this purpose, a list of all RefSeq lncRNAs was retrieved from HGNC BioMart (Table S3) 44 on 29 January 2018 according to the following options; Filter by genes with RefSeqs accession; Status: Approved; Locus group: non‐coding RNA; Locus type: RNA, long non‐coding.

### 2.4 Survival analysis

Prognostic value of those RefSeq lncRNAs that remained in the last step of the data filtration process was investigated using the GEPIA web server. To this end, the prognostic value of all of the remained RefSeq lncRNAs was analysed across the TCGA LUAD dataset with the default options of GEPIA web server. Lastly, lncRNAs with significant prognostic value (Logrank test *P*‐value < 0.05) for OS and/or DFS were selected and named as LUAD Prognostic lncRNAs (LUAD Prognostic lncRNAs = (gene library ⋂ RefSeq‐lncRNAs) ⋂ LUAD survival‐associated lncRNAs). Also, using the GEPIA web server, the expression of LUAD Prognostic lncRNAs (LAProLncRs) was analysed across the LUAD tumour samples compared with normal controls to illustrate their expression dysregulations. Additionally, a multivariate Cox regression analysis with adjustments for the clinicopathological features of patients, including tumour stage, gender and smoking history was done to figure out if any of the prognostic lncRNAs in LUAD could be considered as an independent prognostic factor or not. For this purpose, the Kaplan‐Meier plotter online software (http://kmplot.com/analysis) 45 was used to perform a multivariate Cox regression analysis on a LUAD microarray study (GSE31210). In this step, a microarray dataset rather than an RNA‐Seq one was used to obtain more reliable results.

### 2.5 Coexpression analysis

The coexpression analysis was done for each lncRNA independently. Different resources of TCGA LUAD processed data were integrated in order to identify high‐confident CEGs. To this purpose, first, all of the significantly CEGs with each lncRNA were retrieved from both circlncRNAnet and GEPIA databases and their shared genes were outputted. Then, the intersection of CEGs with the list of all LUAD DEGs was queried ((circlncRNAnet CEGs ⋂ GEPIA CEGs) ⋂ GEPIA DEGs) so as to separate differentially expressed CEGs (DECEGs) (Table S4). Finally, the coexpression networks of lncRNAs with one another and with other DEGs were reconstructed using Cytoscape v3.5.1.^46^

### 2.6 Functional analysis

A coexpression‐based model was applied for the prediction of functional roles of lncRNAs in LUAD. First, the DECEGs of each LAProLncR were used to perform a gene set enrichment analysis for gene ontology‐biological process (GO‐BP) terms via Enrichr web server.^47^, ^48^ It should be noted that because SNHG6 was not significantly differentially expressed in LUAD, not only its DECEGs, but all of its CEGs were used for the gene set enrichment analysis. Then, the FuncPred database^29^ was used to investigate the association of LAProLncRs with GO‐BP terms in normal lung tissue based on the tissue‐specific and evolutionary conserved expression data. Finally, the first ranked GO‐BP terms of Enrichr (according to the highest combined score) and FuncPred (according to the lowest FDR) as well as their intersection were selected as the most remarkable GO‐BP terms and were illustrated as a network using the Cytoscape software. Furthermore, considering the clustered lncRNAs in the lncRNA‐GO‐BP network, the LncPath R package (https://CRAN.R-project.org/package=LncPath) was used to interrogate the synergistic function of lncRNAs across the KEGG pathways. At last, the DECEGs of synergic lncRNAs were mapped onto the predicted pathway using KEGG Mapper^49^ and the resulted pathway was imported into Cytoscape by means of KEGGScape app^50^ and enhanced manually. LncPath conducts a random walk strategy followed by applying a weighted Kolmogorov‐Smirnov statistic to evaluate the pathways related to the lncRNA sets based on their CEGs.

### 2.7 Clinicopathological and demographic analysis

The differential expression of LAProLncRs among different LUAD stages was analysed using the GEPIA web server. Also, the impact of smoking habit and gender on the expression of prognostic lncRNAs in LUAD was investigated using the Lung Cancer Explorer (http://lce.biohpc.swmed.edu/lungcancer). Lung Cancer Explorer is an online database that provides the exploration of gene expression data from several public lung cancer datasets.

### 2.8 Statistical and topological analysis

All of the statistical analyses, except Cox regression analysis, were done using R statistical software (R Development Core Team (2014), freely available at http://www.r-project.org). The multivariate Cox regression analysis was done by the Kaplan‐Meier plotter web server. The Pearson correlation coefficient (*R*)>0.3 was considered as the significant threshold throughout the study. Also, the *P*‐value < 0.05 was considered statistically significant in all of the analyses. In the context of graph topology, two network metrics including betweenness centrality (a measure of node centrality based on shortest paths) and degree (the number of edges incident to each node) were coincidently employed to determine the hub nodes, whenever possible.

## 3 RESULTS

### 3.1 Selection of 168 lncRNAs as the gene library

After filtration of the TCGA LUAD DE‐lncRNAs, only 164 lncRNAs remained. Also, RNA‐seq data analysis resulted in 109 circulating lncRNAs with significant differential abundance (2 < |Log2FC|, adjp < 0.05) in NSCLC plasma samples compared with normal ones (Table S5). Interestingly, all 109 lncRNAs had lower abundance in NSCLC plasma samples compared with normal samples. Filtration of these 109 lncRNAs through circlncRNAnet and cBioPortal databases indicated that four of the 109 circulating lncRNAs were significantly amplified/overexpressed in TCGA LUAD samples (Data not shown). Altogether, 168 lncRNAs were selected for downstream analyses (Table S6).

### 3.2 Presentation of 19 lncRNAs as candidate LUAD biomarkers

**Figure 2:**
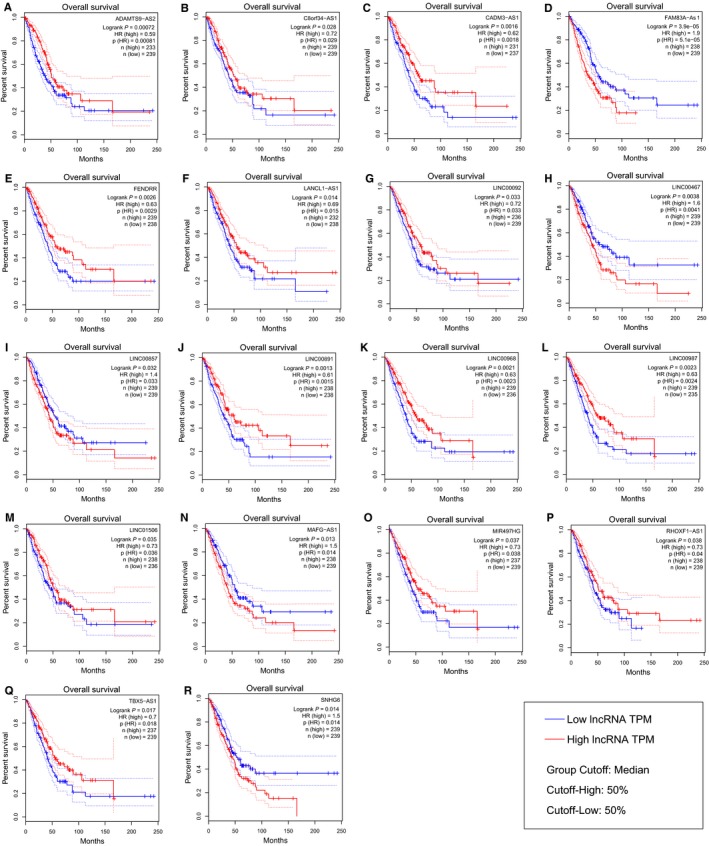
Association of lncRNAs with OS in LUAD. The association of (A) ADAMTS9‐AS2, (B) C8orf34‐AS1, (C) CADM3‐AS1, (D) FAM83A‐AS1, (E) FENDRR, (F) LANCL1‐AS1, (G) LINC00092, (H) LINC00467, (I) LINC00857, (J) LINC00891, (K) LINC00968, (L) LINC00987, (M) LINC01506, (N) MAFG‐AS1, (O) MIR497HG, (P) RHOXF1‐AS1, (Q) TBX5‐AS1, (R) SNHG6, lncRNAs with the OS of LUAD patients. TPM is a unit of transcript expression and the abbreviation of Transcripts per Million. The plots were achieved using GEPIA web server

**Figure 3:**
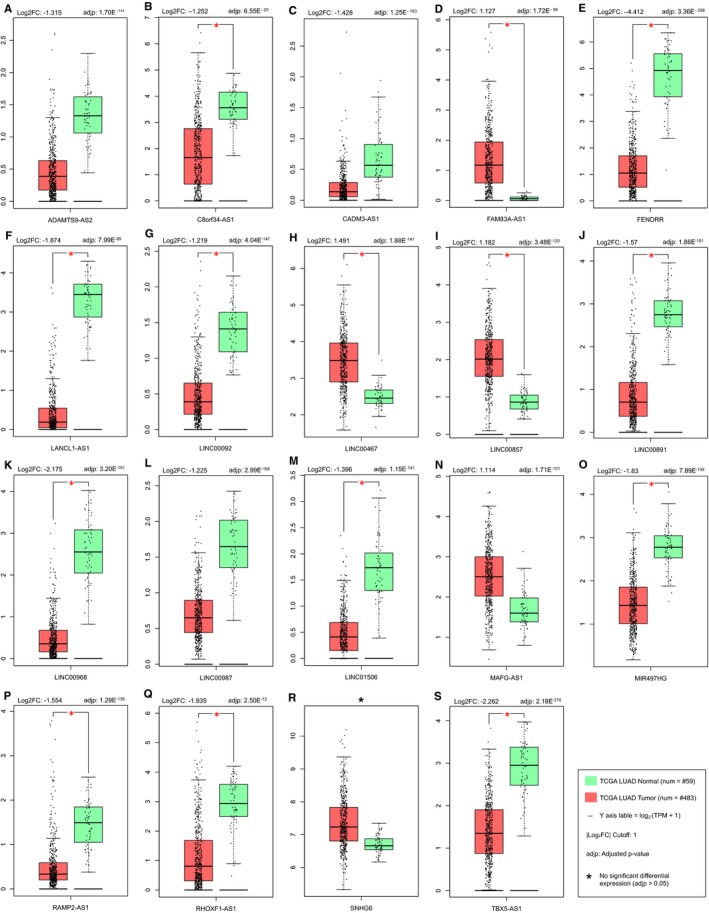
Altered expression of 19 LAProLncRs. The altered expression of (A) ADAMTS9‐AS2, (B) C8orf34‐AS1, (C) CADM3‐AS1, (D) FAM83A‐AS1, (E) FENDRR, (F) LANCL1‐AS1, (G) LINC00092, (H) LINC00467, (I) LINC00857, (J) LINC00891, (K) LINC00968, (L) LINC00987, (M) LINC01506, (N) MAFG‐AS1, (O) MIR497HG, (P) RAMP2‐AS1, (Q) RHOXF1‐AS1, (R) SNHG6, (S) TBX5‐AS1, lncRNAs in LUAD tumour samples. FC and TPM are the abbreviations of Fold‐Change and Transcripts per Million respectively. Box plots were achieved using GEPIA web server

Among all lncRNAs in our gene library, only 62 lncRNAs came out as RefSeq lncRNAs after filtration through HGNC RefSeq lncRNAs (Table S7). Subsequently, survival analyses using the GEPIA web server demonstrated that 19 of the 62 RefSeq lncRNAs had significant prognostic values (Logrank test *P*‐value < 0.05) for LUAD (Table 1). Remarkably, one of these lncRNAs, namely SNHG6, was of the lncRNAs with differential abundance between plasma samples of NSCLC patients and healthy controls. Also, Kaplan‐Meier plots illustrated that the association of these 19 lncRNAs with OS/DFS of patients is in accordance with the dysregulation of these lncRNAs in TCGA LUAD cancer samples (Figure 2 and Figure S1). Actually, while down‐regulated lncRNAs had higher expression levels in patients with higher percentages of OS/DFS, up‐regulated lncRNAs had lower expression levels in those patients. Furthermore, the expression analysis of these lncRNAs using the GEPIA web server demonstrated obvious differences in the expression of these 19 lncRNAs between normal and cancer samples (Figure 3).

**Table 1:** LncRNAs with prognostic value in lung adenocarcinoma (LUAD)

lncRNA symbol	Gene description	Prognostic value	Logrank test *P*‐value
ADAMTS9‐AS2	ADAMTS9 antisense RNA 2	OS	0.00072
C8orf34‐AS1	C8orf34 antisense RNA 1	OS	0.028
CADM3‐AS1	CADM3 antisense RNA 1	OS	0.0016
FAM83A‐AS1	FAM83A antisense RNA 1	DFS	0.0024
	FAM83A antisense RNA 1	OS	3.9e‐05
FENDRR	FOXF1 adjacent non‐coding developmental regulatory RNA	OS	0.0026
LANCL1‐AS1	LANCL1 antisense RNA 1	OS	0.014
LINC00092	long intergenic non‐protein coding RNA 92	OS	0.033
LINC00467	long intergenic non‐protein coding RNA 467	OS	0.0038
LINC00857	long intergenic non‐protein coding RNA 857	OS	0.032
LINC00891	long intergenic non‐protein coding RNA 891	OS	0.0013
LINC00968	long intergenic non‐protein coding RNA 968	OS	0.0021
LINC00987	long intergenic non‐protein coding RNA 987	OS	0.0023
LINC01506	long intergenic non‐protein coding RNA 1506	OS	0.035
MAFG‐AS1	MAFG antisense RNA 1 (head to head)	OS	0.013
MIR497HG	mir‐497‐195 cluster host gene	OS	0.037
RAMP2‐AS1	RAMP2 antisense RNA 1	DFS	0.029
RHOXF1‐AS1	RHOXF1 antisense RNA 1	OS	0.038
	RHOXF1 antisense RNA 1	DFS	0.019
SNHG6	small nucleolar RNA host gene 6	OS	0.014
TBX5‐AS1	TBX5 antisense RNA 1	OS	0.017

Abbreviations: DFS, Disease‐Free Survival; OS, Overall Survival.

### 3.3 LAProLncRs are coexpressed with several other genes

**Figure 4:**
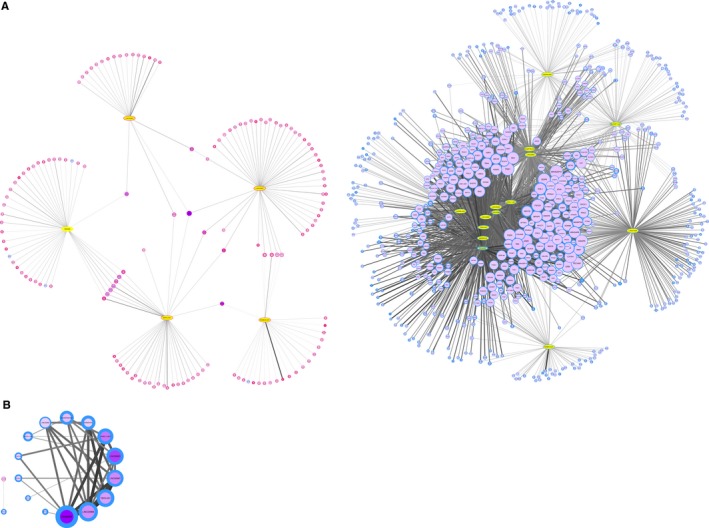
Gene coexpression networks. (A). The network of LAProLncRs and their DECEGs; the yellow nodes represent LAProLncRs. (B) The gene coexpression network of LAProLncRs. The intensity of violet node colour is proportional to betweenness centrality score; stronger violet colour indicates higher betweenness centrality score. Red and Blue node borders are indicative of overexpression and underexpression respectively. The width of node border indicates the GEPIA Log2FC; wider border indicates greater Log2FC. The size of violet nodes and their label font size indicate the node degree; bigger violet node and label font size are indicative of higher node degree. The edge colour and width shows the GEPIA PCC; darker and wider edge is indicative of higher GEPIA PCC. PCC is the abbreviation of Pearson Correlation Coefficient. The networks were reconstructed using the Cytoscape software

The coexpression analysis of lncRNAs indicated that they were significantly coexpressed (PCC > 0.3) with several other DEGs in LUAD (Figure 4A). The coexpression analysis of lncRNAs with other LUAD DEGs also demonstrated that overexpressed and underexpressed lncRNAs do not have common CEGs and tend to cluster in separate modules. According to the topological analysis of the coexpression network, ATIC and JAM2 were identified as the hub nodes in the overexpressed and underexpressed modules respectively. Moreover, 16 of the 19 lncRNAs were significantly coexpressed (PCC > 0.3) with each other of which FENDRR was the one with the highest degree and betweenness centrality in the lncRNAs coexpression network (Figure 4B).

### 3.4 LAProLncRs are involved in several regulatory biological processes

**Figure 5:**
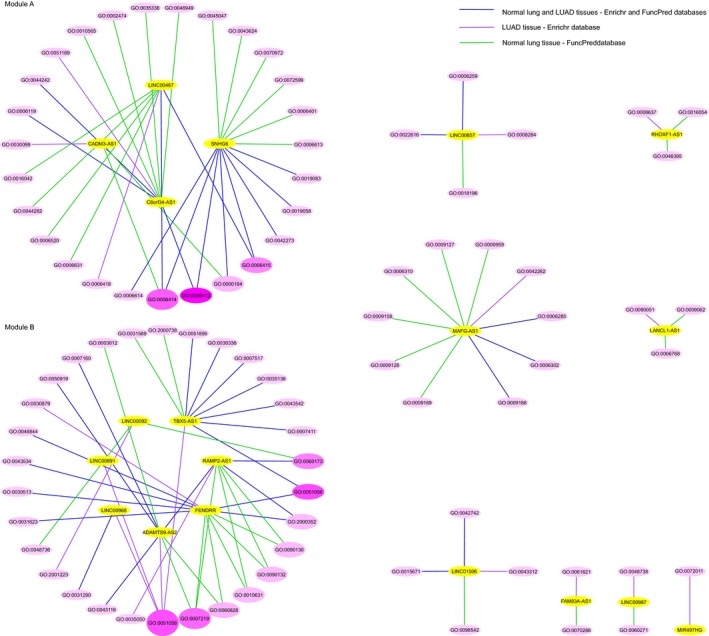
The lncRNA‐GO‐BP association network. The yellow nodes represent LAProLncRs. The intensity of pink node colour indicates the betweenness centrality score; stronger pink colour indicates higher betweenness centrality score. The node height is proportional to node degree; Bigger node indicates higher node degree. The edge colour is representative of the source database of predicted GO term and the reference tissue type. Please refer to Table 2 for finding the description of GO IDs. The network was reconstructed using the Cytoscape software

The gene set enrichment analysis of the DECEGs of LAProLncRs indicated that these lncRNAs might be involved in several regulatory biological processes including cancer‐related ones (Figure 5; Table 2; Table S8). As depicted in the lncRNA‐GO‐BP network, some lncRNAs and their associated GO terms were grouped in modules and might work in common biological processes. While C8orf34‐AS1 and LINC00467 in Module A were mostly associated with the cellular lipid catabolic processes, SNHG6 and CADM3‐AS1 were related to the regulation of protein translation, targeting and localization. On the other hand, lncRNAs in Module B were connected with the biological adhesion processes, cell and tissue migration, apoptosis and signalling pathways including Wnt and Notch signalling pathways. Non‐modulated lncRNAs were associated with cancer‐related biological processes as well; LINC00857 was associated with DNA strand elongation and positive regulation of cell proliferation; MAFG‐AS1 with the regulation of DNA protection, repair and recombination; LINC01506 with the regulation of immune system and responses; RHOXF1‐AS1 with apoptotic and catabolic processes; LANCL1‐AS1 with the regulation of lipid metabolic processes and angiogenesis; MIR497HG with the regulation of endothelium development and migration; and FAM83A‐AS1 and LINC00987 were associated with development and morphogenesis. According to the topological analysis of the lncRNA‐GO‐BP network, GO:0006414 (translational elongation) and GO:0051058 (negative regulation of small GTPase mediated signal transduction) were identified as the hub nodes of Modules A and B respectively. Furthermore, the pathway analysis of Module A and B of the lncRNA‐GO‐BP network demonstrated that the lncRNAs in Module B could have significant synergistic functions (*P*‐value:0.012; FDR:0.024) in the neuroactive ligand‐receptor interaction pathway (Table 3 and Figure S2).

**Table 2:** GO terms associated with LAProLncRs

GO‐BP ID	GO‐BP description
GO:0000184	nuclear‐transcribed mRNA catabolic process, nonsense‐mediated decay
GO:0000959	mitochondrial RNA metabolic process
GO:0002474	antigen processing and presentation of peptide antigen via MHC class I
GO:0003012	muscle system process
GO:0006119	oxidative phosphorylation
GO:0006259	DNA metabolic process
GO:0006285	base‐excision repair, AP site formation
GO:0006302	double‐strand break repair
GO:0006310	DNA recombination
GO:0006401	RNA catabolic process
GO:0006412	translation
GO:0006414	translational elongation
GO:0006415	translational termination
GO:0006418	tRNA aminoacylation for protein translation
GO:0006520	cellular amino acid metabolic process
GO:0006613	cotranslational protein targeting to membrane
GO:0006614	SRP‐dependent cotranslational protein targeting to membrane
GO:0006631	fatty acid metabolic process
GO:0006768	biotin metabolic process
GO:0007160	cell‐matrix adhesion
GO:0007219	Notch signalling pathway
GO:0007411	axon guidance
GO:0007517	muscle organ development
GO:0008284	positive regulation of cell proliferation
GO:0008637	apoptotic mitochondrial changes
GO:0009062	fatty acid catabolic process
GO:0009127	purine nucleoside monophosphate biosynthetic process
GO:0009128	purine nucleoside monophosphate catabolic process
GO:0009158	ribonucleoside monophosphate catabolic process
GO:0009168	purine ribonucleoside monophosphate biosynthetic process
GO:0009169	purine ribonucleoside monophosphate catabolic process
GO:0010565	regulation of cellular ketone metabolic process
GO:0010631	epithelial cell migration
GO:0015671	oxygen transport
GO:0016042	lipid catabolic process
GO:0016054	organic acid catabolic process
GO:0018196	peptidyl‐asparagine modification
GO:0019058	viral life cycle
GO:0019083	viral transcription
GO:0022616	DNA strand elongation
GO:0030099	myeloid cell differentiation
GO:0030336	negative regulation of cell migration
GO:0030513	positive regulation of BMP signalling pathway
GO:0030879	mammary gland development
GO:0031290	retinal ganglion cell axon guidance
GO:0031589	cell‐substrate adhesion
GO:0031623	receptor internalization
GO:0035050	embryonic heart tube development
GO:0035136	forelimb morphogenesis
GO:0035338	long‐chain fatty‐acyl‐CoA biosynthetic process
GO:0042262	DNA protection
GO:0042273	ribosomal large subunit biogenesis
GO:0042742	defense response to bacterium
GO:0043116	negative regulation of vascular permeability
GO:0043312	neutrophil degranulation
GO:0043534	blood vessel endothelial cell migration
GO:0043542	endothelial cell migration
GO:0043624	cellular protein complex disassembly
GO:0044242	cellular lipid catabolic process
GO:0044282	small molecule catabolic process
GO:0045047	protein targeting to ER
GO:0046395	carboxylic acid catabolic process
GO:0046949	fatty‐acyl‐CoA biosynthetic process
GO:0048736	appendage development
GO:0048738	cardiac muscle tissue development
GO:0048844	artery morphogenesis
GO:0050919	negative chemotaxis
GO:0051056	regulation of small GTPase mediated signal transduction
GO:0051058	negative regulation of small GTPase mediated signal transduction
GO:0051189	prosthetic group metabolic process
GO:0051895	negative regulation of focal adhesion assembly
GO:0060173	limb development
GO:0060271	cilium morphogenesis
GO:0060828	regulation of canonical Wnt signalling pathway
GO:0061621	canonical glycolysis
GO:0070286	axonemal dynein complex assembly
GO:0070972	protein localization to endoplasmic reticulum
GO:0072011	glomerular endothelium development
GO:0072599	establishment of protein localization to endoplasmic reticulum
GO:0090051	negative regulation of cell migration involved in sprouting angiogenesis
GO:0090130	tissue migration
GO:0090132	epithelium migration
GO:0098542	defense response to other organism
GO:2000352	negative regulation of endothelial cell apoptotic process
GO:2000738	positive regulation of stem cell differentiation
GO:2001223	negative regulation of neuron migration

Abbreviations: BP, Biological Process; GO, Gene Ontology.

**Table 3:** LAProLncRs and their coexpressed genes with synergistic function in the neuroactive ligand‐receptor interaction pathway

DECEG	LncRNA symbol	PCC	Dysregulation in LUAD
CHRM1	FENDRR	0.9	Down‐regulated
ADRA1A	ADAMTS9‐AS2	0.54	Down‐regulated
ADRB2	ADAMTS9‐AS2	0.59	Down‐regulated
ADRB1	FENDRR	0.76	Down‐regulated
ADRB2	FENDRR	0.83	Down‐regulated
ADRB2	LINC00092	0.58	Down‐regulated
ADRB2	LINC00891	0.65	Down‐regulated
ADRB2	LINC00968	0.76	Down‐regulated
EDNRB	ADAMTS9‐AS2	0.56	Down‐regulated
EDNRB	FENDRR	0.92	Down‐regulated
EDNRB	TBX5‐AS1	0.73	Down‐regulated
EDNRB	LINC00092	0.59	Down‐regulated
EDNRB	LINC00891	0.6	Down‐regulated
EDNRB	LINC00968	0.79	Down‐regulated
NMUR1	FENDRR	0.88	Down‐regulated
NMUR1	LINC00092	0.65	Down‐regulated
NMUR1	LINC00968	0.69	Down‐regulated
NMUR1	RAMP2‐AS1	0.4	Down‐regulated
NMUR1	TBX5‐AS1	0.74	Down‐regulated
PTGIR	TBX5‐AS1	0.79	Down‐regulated
S1PR1	ADAMTS9‐AS2	0.58	Down‐regulated
S1PR1	FENDRR	0.87	Down‐regulated
S1PR1	LINC00092	0.58	Down‐regulated
S1PR1	LINC00968	0.79	Down‐regulated
RXFP1	FENDRR	0.88	Down‐regulated
RXFP1	RAMP2‐AS1	0.4	Down‐regulated
RXFP1	LINC00968	0.74	Down‐regulated
CALCRL	FENDRR	0.93	Down‐regulated
CALCRL	TBX5‐AS1	0.74	Down‐regulated
CALCRL	LINC00092	0.58	Down‐regulated
CALCRL	LINC00968	0.73	Down‐regulated
VIPR1	FENDRR	0.91	Down‐regulated
GRIA1	ADAMTS9‐AS2	0.59	Down‐regulated
GRIK4	ADAMTS9‐AS2	0.53	Down‐regulated
GRIA1	FENDRR	0.82	Down‐regulated
GRIA1	TBX5‐AS1	0.78	Down‐regulated
GRIK4	TBX5‐AS1	0.72	Down‐regulated
GRIA1	LINC00092	0.67	Down‐regulated
GRIK4	LINC00092	0.58	Down‐regulated
GRIA1	LINC00891	0.66	Down‐regulated
GRIK4	LINC00891	0.59	Down‐regulated
GRIA1	LINC00968	0.84	Down‐regulated
GRIK4	LINC00968	0.74	Down‐regulated

Abbreviations: DECEGs, differentially expressed coexpressed genes; LUAD, lung adenocarcinoma; PCC, Pearson correlation coefficient.

### 3.5 The expression of some of the LAProLncRs is associated with clinicopathological and demographic features

**Figure 6:**
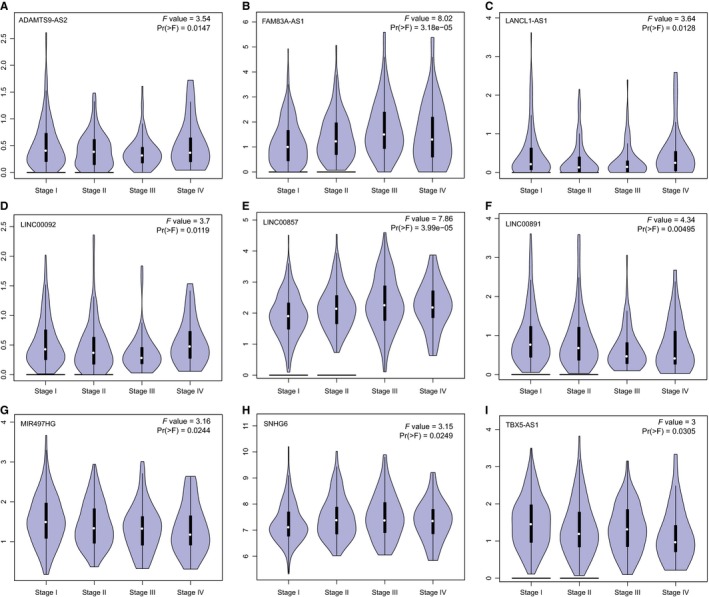
The expression‐stage plot of LAProLncRs. (A) The expression‐stage plot of ADAMTS9‐AS2 lncRNA. (B) The expression‐stage plot of FAM83A‐AS1 lncRNA. (C) The expression‐stage plot of LANCL1‐AS1 lncRNA. (D) The expression‐stage plot of LINC00092 lncRNA. (E) The expression‐stage plot of LINC00857 lncRNA. (F) The expression‐stage plot of LINC00891 lncRNA. (G) The expression‐stage plot of MIR497HG lncRNA. (H) The expression‐stage plot of SNHG6 lncRNA. (I) The expression‐stage plot of TBX5‐AS1 lncRNA. The plots were achieved by the GEPIA web server

**Figure 7:**
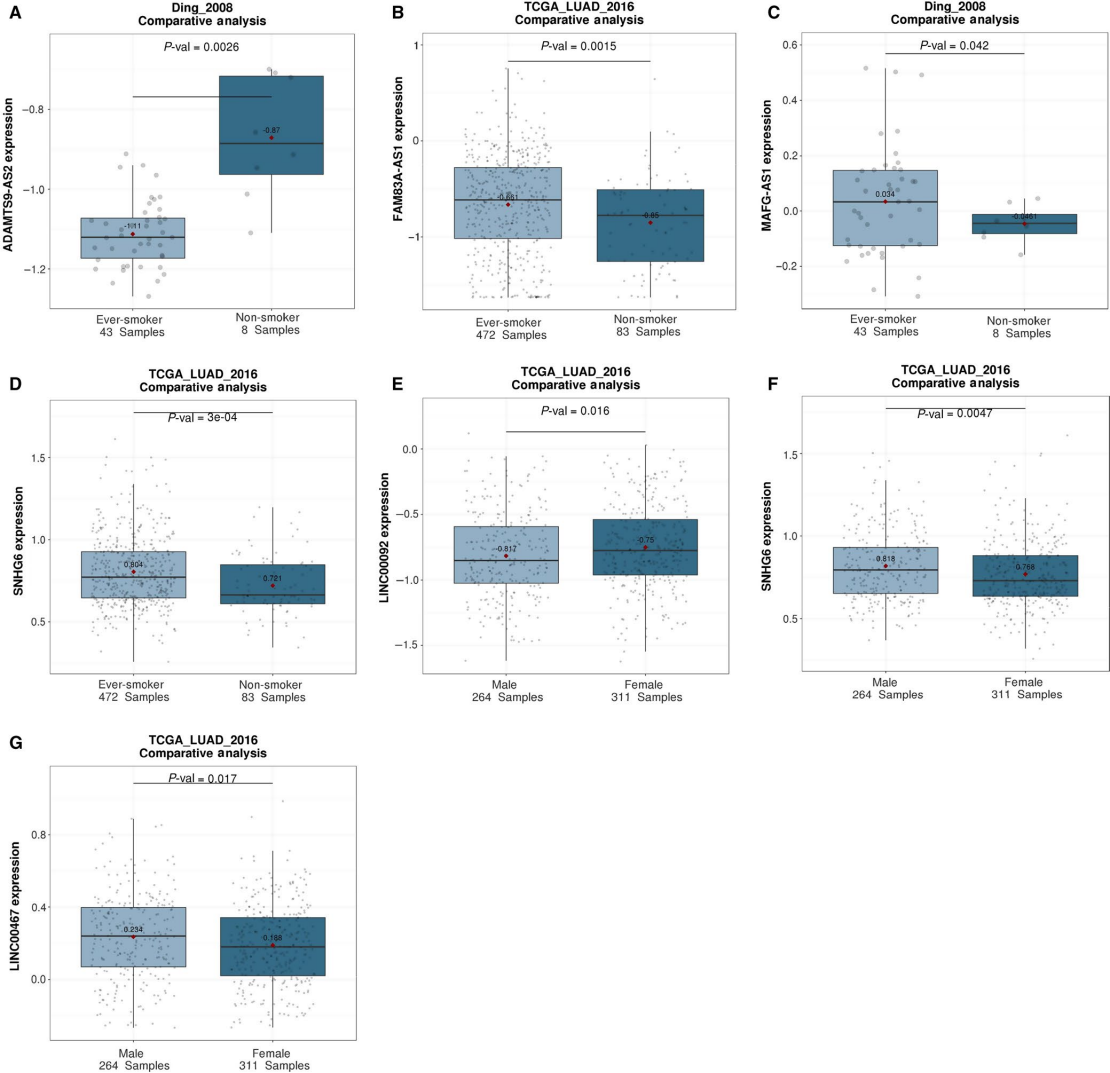
The impact of smoking habit and gender on the expression of LAProLncRs. (A) The impact of smoking habit on ADAMTS9‐AS2 expression in LUAD. (B) The impact of smoking habit on FAM83A‐AS1 expression in LUAD. (C) The impact of smoking habit on MAFG‐AS1 expression in LUAD. (D) The impact of smoking habit on SNHG6 expression in LUAD. (E) The impact of gender on LINC00092 expression in LUAD. (F) The impact of gender on SNHG6 expression in LUAD. (G) The impact of gender on LINK00467 expression in LUAD. The plots were obtained from the Lung Cancer Explorer

The analyses demonstrated that the expression of nine of the 19 LAProLncRs was significantly associated (*P*‐value < 0.05) with different stages of LUAD (Figure 6). Also, the violin plots in Figure 6 indicated that the association of these nine lncRNAs with different stages of LUAD is in accordance with the dysregulation of these lncRNAs in TCGA LUAD cancer samples; while up‐regulated lncRNAs usually had higher expression levels in patients with higher tumour stages, down‐regulated lncRNAs had lower expression levels in those patients. Likewise, the expression of four of the 19 LAProLncRs was significantly influenced (*P*‐value < 0.05) by the smoking habit in LUAD and this association was in accordance with the dysregulation of these lncRNAs in TCGA LUAD cancer samples (Figure 7A‐D). Furthermore, the expression of three of the 19 LAProLncRs was significantly associated (*P*‐value < 0.05) with the gender of LUAD patients (Figure 7E‐G). However, multivariate Cox regression analyses indicated that while the expression level of most of the LAProLncRs and the stage of patients are independent prognostic factors, gender and smoking history do not have independent prognostic value for LUAD (Table 4).

**Table 4:** Prognostic value of LAProLncRs with adjustments for clinicopathological features of patients

LncRNA	Dysregulation (Up/Down)	Overall survival^a^	Multivariate analysis			
			Covariate	HR	95%CI	*P*‐value
ADAMTS9‐AS2	Down	Lower	LncRNA expression	0.23	0.11‐0.5	0.0002
			Stage	2.8	1.39‐5.63	0.0039
			Gender	1.1	0.42‐2.87	0.8426
			Smoking history	0.9	0.34‐2.38	0.832
FENDRR	Down	Lower	LncRNA expression	0.1	0.01‐0.74	0.0242
			Stage	3.42	1.73‐6.73	0.0004
			Gender	1.07	0.44‐2.58	0.8819
			Smoking history	0.93	0.38‐2.28	0.88
LINC00092	Down	Lower	LncRNA expression	0.26	0.11‐0.62	0.0022
			Stage	3.4	1.71‐6.77	0.0005
			Gender	1.19	0.46‐3.08	0.7208
			Smoking history	1.15	0.43‐3.09	0.7758
LINC00467	Up	Higher	LncRNA expression	0.42	0.17‐1.03	0.058
			Stage	3.6	1.81‐7.16	0.0003
			Gender	1.07	0.43‐2.66	0.8839
			Smoking history	0.72	0.29 ‐ 1.84	0.4969
LINC00857	Up	Higher	LncRNA expression	2.78	1.4‐5.51	0.0034
			Stage	3.74	1.9‐7.38	0.0001
			Gender	1.17	0.45‐3.06	0.7474
			Smoking history	0.9	0.34‐2.39	0.8268
LINC00968	Down	Lower	LncRNA expression	0.29	0.12‐0.7	0.0055
			Stage	2.6	1.27‐5.34	0.0092
			Gender	1.22	0.5‐2.97	0.6559
			Smoking history	1.07	0.43‐2.66	0.8775
MAFG‐AS1	Up	Higher	LncRNA expression	1.71	0.8‐3.68	0.1693
			Stage	4.18	2.12‐8.23	0
			Gender	1.05	0.42‐2.61	0.9143
			Smoking history	0.75	0.3‐1.9	0.5491
MIR497HG	Down	Lower	LncRNA expression	0.62	0.32‐1.22	0.1637
			Stage	3.75	1.9‐7.42	0.0001
			Gender	1.15	0.45‐2.92	0.7695
			Smoking history	0.82	0.32‐2.09	0.6722
RAMP2‐AS1	Down	Lower	LncRNA expression	0.33	0.15‐0.73	0.006
			Stage	3.6	1.82‐7.12	0.0002
			Gender	1.24	0.48‐3.23	0.6542
			Smoking history	0.91	0.34‐2.39	0.8417
SNHG6	Up^b^	Higher	LncRNA expression	0.52	0.26‐1.04	0.0638
			Stage	3.88	1.97‐7.65	0.0001
			Gender	1.25	0.5‐3.14	0.6297
			Smoking history	0.87	0.34‐2.19	0.7626
TBX5‐AS1	Down	Lower	LncRNA expression	0.21	0.09‐0.49	0.0004
			Stage	2.57	1.27‐5.19	0.0086
			Gender	1.32	0.52‐3.34	0.5632
			Smoking history	1.13	0.43‐2.93	0.8041

Abbreviations: CI, Confidence Interval; HR, Hazard Ratio.

a Expression level of the lncRNA in LUAD patients with lower overall survival (OS) compared to patients with higher OS.

b Higher expression in tumour vs normal samples but with no significant differential expression.

## 4 DISCUSSION AND CONCLUSION

The aim of this investigation was to systematically examine the diagnostic and predictive value of lncRNAs for LUAD and to annotate their functions by employing a bioinformatic and systems biology approach. Recently, the focus of cancer investigations has shifted from protein‐coding genes to non‐coding transcripts especially lncRNAs given their diverse regulatory roles on gene expression at the transcriptional and post‐transcriptional levels. Although the association of tens of lncRNAs with LUAD has been previously reported, most of them are not annotated and their functions in LUAD development have not been deciphered. Dysregulation of eight of the 19 LAProLncRs including ADAMTS9‐AS2,^51^ FENDRR,^52^ LINC00968,^53^ RAMP2‐AS1,^54^ SNHG6,^55^ LINC00092,^22^ FAM83A‐AS1^22^ and TBX5‐AS1^56^ in LUAD has been previously demonstrated. Though, the prognostic value of most of these lncRNAs for LUAD patients has not been evaluated. Similarly, the differential abundance of lncRNAs in the plasma of normal and LUAD specimens, which could be used as signatures for diagnosis and prognosis of LUAD, has not yet been examined. Altogether, the detection and characterization of LAProLncRs could help early diagnosis, prognosis and treatment of patients with this deadly disease. In this study, we addressed all of the above issues.

Circulating lncRNAs have recently emerged as novel cancer biomarkers for diagnostic and prognostic purposes. For instance, the circulating lncRNAs NEAT1, ANRIL and SPRY4‐IT1 have been suggested as new diagnostic biomarkers for NSCLC.^5^ It has been reported that the dysregulation of lncRNAs in plasma is in accordance with their dysregulation in the source tumour tissue.^16^ Thus, according to our RNA‐Seq data analysis, dysregulated lncRNAs in NSCLC plasma samples could be used for diagnostic purposes and might play important roles in NSCLC development. However, there is a delicate point that is yet ignored regarding the abundance of circulating RNAs. Circulating RNAs originate as a result of different cellular events especially apoptosis and escape from the enzymatic degradation via absorption by extracellular vesicles including apoptotic bodies.^57^, ^58^ Also, it is well known that cancer cells evade apoptosis by employing different strategies.^59^ Consequently, circulating RNAs might have a lower abundance in the plasma of cancer specimens relative to normal ones. Altogether, further studies on the diagnostic and prognostic value of all 109 circulating lncRNAs might represent new potential circulating lncRNA biomarkers as tools for early diagnosis, prognosis and monitoring of treatment response for NSCLC patients. According to our results, one of the 19 LAProLncRs, namely SNHG6, was also dysregulated in NSCLC plasma samples and could possibly be used as a diagnostic and/or prognostic biomarker for non‐invasive detection and treatment monitoring of LUAD. In addition, other 18 LAProLncRs could be used as diagnostic and/or prognostic biomarkers in clinical practice as well. Notably, the association of six of the 19 LAProLncRs with lung cancer has been previously confirmed by microarray studies (Table 5). However, there is no report about the other 13 LAProLncRs.

**Table 5:** The association of six lncRNAs with lung cancer based on microarray studies

lncRNA symbol	Cancer subtype	Methods	Reference
ADAMTS9‐AS2	NSCLC	Microarray	60
FENDRR	LUAD	qRT‐PCR and RNA‐FISH	52
	NSCLC	Microarray	60
	LUSC	RNA‐seq and Microarray	33
LINC00857	Lung cancer	Microarray	61
LINC00968	NSCLC	Microarray	60
	LUSC	RNA‐seq and Microarray	33
LINC00987	LUAD	Microarray	62
MAFG‐AS1	NSCLC	Microarray	60

Abbreviations: NSCLC: Non‐Small Cell Lung Cancer; LUSC: Lung Squamous Cell carcinoma; LUAD: Lung Adenocarcinoma; qRT‐PCR: Quantitative Reverse Transcription‐Polymerase Chain Reaction; RNA‐FISH: Fluorescent In situ Hybridization Targeting Ribonucleic Acid Molecules

Considering the guilt by association principle, CEGs might share common features. Although application of this principle could readily help prediction of functions and features of unknown genes in normal cells and tissues, employing this principle to annotate unknown genes in diseased cells is somewhat challenging. Regarding the association of genes and cancer progression, non‐differentially expressed genes might not play an active and direct role in the development of tumours. In other words, DE‐lncRNAs have common features with their DECEGs and not all of their CEGs. This is a key point that has been ignored in almost all of the studies by far, which may mislead the authors and result in incorrect outcomes and interpretations. Accordingly, we only considered those CEGs of LAProLncRs that were differentially expressed as well, namely DECEGs, in all of the analyses. The results of coexpression analyses illustrate that the overexpressed and underexpressed LAProLncRs are independently clustered with their DECEGs in two big modules. This implies that overexpressed and underexpressed LAProLncRs are involved in different biological processes and networks. Also, hub nodes in the coexpression networks might be driving cancer genes and potential drug targets for the treatment of LUAD. The genes *ATIC* and *JAM2* were identified as the hub nodes in the overexpressed and underexpressed LAProLncRs modules respectively. According to the IGDB.NSCLC database,^63^ the dysregulation of ATIC and JAM2 in LUAD is confirmed by microarray studies as well. *ATIC* can be translocated with *ALK*, a potential target for the treatment of NSCLC.^64^ Also, Pemetrexed, an approved drug for unresectable and metastatic non‐squamous NSCLC, is an antifolate that inhibits the products of *ATIC* and some other genes.^65^ Also, polymorphisms in *ATIC*, rs12995526 for instance, could impact on the therapeutic efficacy of Pemetrexed‐treated patients with LUAD.^66^ Moreover, inhibition of ATIC or its knockdown by small interfering‐RNA (siRNA) is a novel chemoradiosensitization strategy which might enhance the treatment efficacy of LUAD patients.^67^ JAM2 is a multifunctional transmembrane protein and is involved in the regulation of diverse cellular processes such as cell growth, proliferation, angiogenesis and tumour metastasis. It is reported that JAM2 is down‐regulated in NSCLC^68^ and LUAD.^69^ Also, Tian et al demonstrated that JAM2, ADARB1, FENDRR and some other LUAD DEGs might synergistically function in the tumourigenesis of stage I LUAD.^70^ Furthermore, Glen et al reported that JAM2 could be targeted for the treatment of NSCLC.^71^ In addition, according to the topological features of the coexpression network of LAProLncRs with each other, FENDRR is the most important node and might essentially contribute to the tumourigenesis of LUAD.

The gene set enrichment analysis demonstrated that most of the LAProLncRs and their associated GO‐BP terms are clustered in two modules. The LAProLncRs in Module A are mostly related to protein and lipid regulatory processes especially lipid catabolic processes. Specific lipids play important roles in endoplasmic reticulum stress, intracellular oncogenic signalling and the relation between cancer cells and cells of the tumour microenvironment.^72^ Also, it has been shown that the aberrant lipid metabolism promotes prostate cancer^73^ and blocking of the lipid catabolism decreases prostate tumour growth.^72^ In addition, GO:0006414 (translational elongation) is the hub GO‐BP term in Module A and is common among CADM3‐AS1, LINC00467 and SNHG6. Translation elongation factors play significant roles in cancer development in a cancer‐specific manner. Also, their overexpression predicts poor prognosis in lung cancer.^74^ The LAProLncRs in Module B of the lncRNA‐GO‐BP network are associated with well‐known cancer‐related biological processes and signalling pathways and GO:0051058 (negative regulation of small GTPase mediated signal transduction) was identified as the hub GO‐BP term in this module. Members of the Rab family of small GTPase superfamily are essential factors in tumourigenesis^75^ and their up‐regulation is associated with poor prognosis and aggressiveness of lung, breast, ovarian, renal and other cancers.^76^ Actually, they play essential roles in the regulation of metabolism, cell‐cell adhesion and cell proliferation and migration,^77^ which are concordant with other GO‐BP terms in Module B. Non‐modulated LAProLncRs are connected with cancer‐related GO‐BP terms as well. These data provide insights into the functional roles of LAProLncRs in LUAD tumourigenesis. In addition to hub GO‐BP terms, the shared terms between LUAD and normal lung tissues (blue edges in Figure 5) should also be considered with a higher priority in future studies. Also, the pathway enrichment analysis demonstrated that the LAProLncRs in Module B might synergistically function in the neuroactive ligand‐receptor interaction pathway. The neuroactive ligand‐receptor interaction is a methylation‐enriched pathway^78^ which its association with LUSC,^79^ osteosarcoma,^80^ breast cancer,^81^ colon cancer,^82^ pancreatic cancer^83^ and hepatocellular carcinoma^78^ has been previously reported. However, further studies are required to decode the precise functional roles of Module B LAProLncRs in this pathway as well as the association of this pathway with LUAD.

The clinicopathological and demographic analyses indicated that the expression level of some of the LAProLncRs is associated with cancer stage, sex and smoking habits in LUAD patients. Besides, the tumour stage is negatively correlated with survival period of NSCLC patients.^84^ This implies that the expression level of LAProLncRs could be used as an additional signature for distinguishing between different stages and consequently predicting the survival period of LUAD patients. Also, results of the association analysis of smoking habit with the expression of LAProLncRs are consistent with the results of differential expression analysis. Actually, while down‐regulated LAProLncRs have a lower expression level in smoker LUAD patients, up‐regulated LAProLncRs have a higher expression level in such patients compared with non‐smoker LUAD patients. Furthermore, based on the results of demographic analyses, men might be more vulnerable to LUAD. On the other hand, adjustment of the survival analysis of the expression of LAProLncRs for clinicopathological features of LUAD patients demonstrated that while gender and smoking history are not independent prognostic factors, tumour stage of the patients and the expression level of most of the LAProLncRs have independent prognostic value in LUAD (Table 4).

Collectively, we conducted the most comprehensive systematic analysis and functional annotation, by far, on the prognostic lncRNAs of LUAD and presented 19 lncRNAs as novel LAProLncRs. Several novel biomarkers and drug targets were suggested which might open up new avenues for the early diagnosis, prognosis and treatment of LUAD patients. Also, our research lays the groundwork for the design of the next studies. However, we faced several limitations in this study that should be noticed in future studies. As we used available online tools with default options in several steps of the project, investigation of the expression level and coexpression of LAProLncRs and their CEGs in different contexts such as age, gender, smoking habit and tumour stage and simultaneous consideration of all of these conditions was not possible. Also, the number of normal and NSCLC plasma samples that we had access to was too low and consequently our results regarding the differential abundance of lncRNAs in the blood might not be robust enough. Additionally, further in silico, in vitro and in vivo assays are required to evaluate the potential of LAProLncRs as biomarkers and/or drug targets for LUAD patients.

## CONFLICT OF INTEREST

The authors have no conflict of interest.

## AUTHOR CONTRIBUTIONS

AS: Designed the bioinformatic and systems biology analyses, performed all of the analyses and wrote the first manuscript draft. ZR: Supervised the whole study and revised the final version of the manuscript. AN: Designed the bioinformatic and systems biology analyses, supervised the whole study and revised the final version of the manuscript. All authors read and approved the final manuscript.

## Data Availability

All datasets generated/analysed for this study are included in the manuscript and the supplementary files.
