# Supplementary material for: Survival analysis and functional annotation of long non‐coding RNAs in lung adenocarcinoma
Source: J Cell Mol Med. 2019 Jun 18;23(8):5600–17. doi: 10.1111/jcmm.14458 (PMC6652661; doi:10.1111/jcmm.14458)
Supplement: Supplementary file 1 [file JCMM-23-5600-s001.pdf]

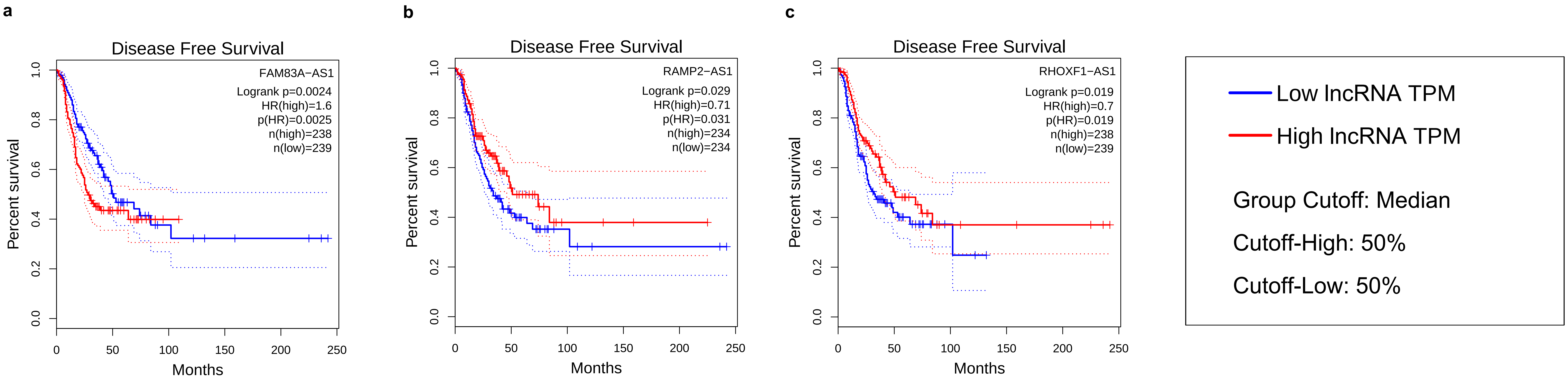

Supplementary Figure S1. Association of lncRNAs with DFS in LUAD. a. The association of FAM83A−AS1 lncRNA with the DFS of LUAD patients. b. The association of RAMP2−AS1 lncRNA with the DFS of LUAD patients. c. The association of RHOXF1−AS1 lncRNA with the DFS of LUAD patients. TPM is a unit of transcript expression and the abbreviation of Transcripts Per Million. The plots were achieved using GEPIA web server.
