# Supplementary material for: Survival analysis and functional annotation of long non‐coding RNAs in lung adenocarcinoma
Source: J Cell Mol Med. 2019 Jun 18;23(8):5600–17. doi: 10.1111/jcmm.14458 (PMC6652661; doi:10.1111/jcmm.14458)
Supplement: Supplementary file 2 [file JCMM-23-5600-s002.pdf]

Pathway title:Neuroactive ligand-receptor interaction

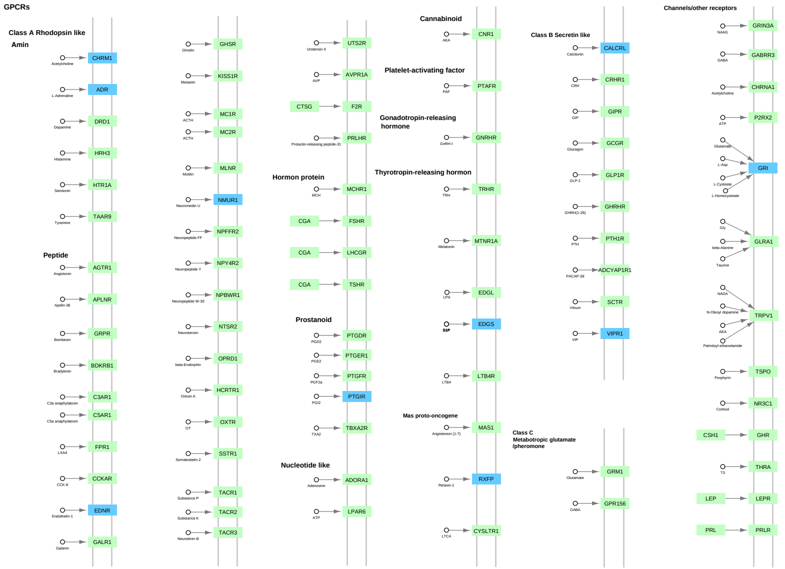

Supplementary Figure S2. The neuroactive ligand-receptor interaction KEGG pathway. The blue nodes represent the DECEGs of lncRNAs which were mapped onto the pathway. The pathway was downloaded from the KEGG database, imported into the Cytoscape by means of KEGGScape app and was enhanced manually. DECEGs is the abbreviation of Differentially Expressed Coexpressed Genes.
